# Molecular changes tracking through multiscale fluorescence microscopy differentiate Meningioma grades and non-tumoral brain tissues

**DOI:** 10.1038/s41598-020-78678-4

**Published:** 2021-02-15

**Authors:** Hussein Mehidine, Matthieu Refregiers, Frédéric Jamme, Pascale Varlet, Marjorie Juchaux, Bertrand Devaux, Darine Abi Haidar

**Affiliations:** 1 Université Paris-Saclay, CNRS/IN2P3, IJCLab, 91405 Orsay, France; 2 Université de Paris, IJCLab, 91405 Orsay, France; 3 DISCO Beamline, Synchrotron SOLEIL, 91192 Gif-sur-Yvette, France; 4 GHU Psychiatrie et Neurosciences, site Sainte-Anne, service de neuropathologie, 75014 Paris, France; 5 IMA BRAIN, INSERM U894, Centre de Psychiatrie Et de Neurosciences, 75014 Paris, France; 6 Université de Paris, 75006 Paris, France

**Keywords:** Biological fluorescence, Cancer imaging, Cancer metabolism, Cancer microenvironment, Tumour biomarkers, Medical research, Oncology, Optics and photonics

## Abstract

Meningioma is the most common primary intracranial extra-axial tumor. Total surgical removal is the standard therapeutic method to treat this type of brain tumors. However, the risk of recurrence depends on the tumor grade and the extent of the resection including the infiltrated dura mater and, if necessary, the infiltrated bone. Therefore, proper resection of all invasive tumor borders without touching eloquent areas is of primordial in order to decrease the risk of recurrence. Nowadays, none of the intraoperative used tools is able to provide a precise real-time histopathological information on the tumor surrounding areas to help the surgeon to achieve a gross total removal. To respond to this problem, our team is developing a multimodal two-photon fluorescence endomicroscope, compatible with the surgeon tool, to better delimitate tumor boundaries, relying on the endogenous fluorescence of brain tissues. In this context, we are building a tissue database in order to specify each brain tissue, whether healthy or tumoral, with its specific optical signature. In this study, we present a multimodal and multiscale optical measurements on non-tumoral control brain tissue obtained in epilepsy surgery patients and several meningioma grades. We investigated tissue auto-fluorescence to track the molecular changes associated with the tumor grade from deep ultra-violet (DUV) to near infrared (NIR) excitation. Micro-spectroscopy, fluorescence lifetime imaging, two-photon fluorescence imaging and Second Harmonic Generation (SHG) imaging were performed. Several optically derived parameters such as collagen crosslinks fluorescence in DUV, SHG emission in NIR and long lifetime intensity fraction of Nicotinamide Adenine Dinucleotide and Flavins were correlated to discriminate cancerous tissue from control one. While collagen response managed to discriminate meningioma grades from control samples with a 100% sensitivity and 90% specificity through a 3D discriminative algorithm.

## Introduction

Being one of the most common cancer that affects humans, Central Nervous System (CNS) tumors are one of the deadliest with a very low survival rate compared with other cancer types^1^. Numerous types of CNS tumors exist which are classified into different categories referring to their originating cell and to their pathological class^2^. Meningioma, known also as meningeal tumors, are one of the most frequently diagnosed primary CNS tumor, presenting more than 30% of all CNS tumor cases^1,3^. In the last edition of WHO classification of CNS tumors (2016)^2^, meningiomas were classified into three major groups which differ in grade and biological behavior: Grade I, a benign tumor recognized by the absence of anaplastic features; Grade II, an atypical tumor known by its invasive development towards brain, surrounding dura matter and that sometimes reaches the skull bone; and Grade III, a rare incident tumor with about 1–6% of the diagnosed meningiomas^4^, known as an anaplastic tumor and the most malignant grade known by its agressive subtypes and a very high rate of mitoses^5^.

These aggressive subtypes expose faster tumor progression, invasiveness and recurrence^6^. However, even benign meningiomas commonly invade adjacent anatomical structures, especially the dura matter, although the rate and extent of local spread are often greater in the more aggressive subtypes^4^. Thus, depending on location and grade, some benign meningioma types can become deadly and fatal, due to their progressive growth in the skull, if they are not treated or excised quickly^1,4^.

For initial diagnosis, contrast CT scan, MRI with gadolinium contrast are the modalities of choice, due to the fact that meningiomas are extra-axial and present a dense tissue structure.

During surgery, the neurosurgeon aims to remove the entire solid tumor detected by MRI. However, recurrence rate has been shown to correlate with the extent of tumor resection^6,7^. This correlation was presented by Donald Simpson which described a grading system that has been validated for recurrence rate prediction in grade I-III meningioma^8^.

To improve the resection quality, many intraoperative techniques have been implemented to help the surgeons with their work such as Intraoperative MRI with neuro-navigation^9^ or operating microscope^10^, which offers a global view of the surgical cavity with a clear visualization of anatomical structures.

However, these techniques can’t offer sufficient information on the histopathological nature of the examined region on a subcellular scale, or to provide a real-time multiple information on dural infiltration and extrinsic brain invasion^11^. However, the histopathological analysis, based on Hematoxylin and Eosin (H&E) staining, is still the standard technique used to acquire a definite diagnosis. However, this analysis intervenes after the operation and takes several days to provide a definite diagnosis.

On the other side, advanced optical imaging techniques such as confocal fluorescence microscopy and optical coherence tomography (OCT) presented advanced bio-imaging capabilities with subcellular scale resolution for in vivo imaging applications such as optical biopsy or brain imaging in animals^12,13^. In addition, fluorescence imaging acquired through two-Photon excitation has been shown to provide high-resolution imaging with lower phototoxicity and deeper penetration depth in tissues^12,13^.

Fluorescence spectroscopy of cancerous tissues started to be investigated many years ago. R. R. Alfano and his group reported several studies on rat kidney, human breast, lungs, uterus, ovarian and cervix cancer tissues^14–17^. where changes in fluorescence spectral emission and fluorescence lifetimes between healthy and cancerous tissues were reported. Later after, tracking the endogenous fluorescence of several endogenous fluorophores as well their fluorescence lifetime such as Nicotinamide Adenine Dinucleotide (NADH) and the Flavins (FAD) tissues has been widely developed, in order to discriminate tumoral, healthy and infiltrated tissues^18–22^. The autofluorescence of these fluorophores and their related metabolic ratios has been widely studied to track and to provide reliable information on tissue metabolism^18–21^. They have been shown as an efficient indicator of healthy and tumoral tissues in bladder^23^, breasts^24^, and brain tissues^21,25^.

Excitation in deep UV (DUV), efficient to expose the auto-fluorescence of bio-macromolecules such as tryptophan and collagen crosslinks, was also reported to provide an additional marker for monitoring cellular and metabolic status^26,27^. It highlights the fluorescence of several fluorophores that do not fluoresce if excited in visible or NIR^27^ which constitute a good base to correlate the auto-fluorescence of all these fluorophores. While collagen crosslinks fluoresce in DUV, the collagen polymers are a strong source of Second Harmonic Generation (SHG) signal when using NIR excitation, indicating the presence of vascularized structures. So, using NIR excitation wavelength and correlating with DUV excitation, the ability to relate the fluorescence response of this molecule with its SHG emission will improve the robustness of our markers and the determination of the metabolic alteration and the structural variation in cancerous tissues.

To this end, and to answer the neurosurgeon needs, a two-photon fluorescence endomicroscope is under development. It is able to collect and analyze the autofluorescence of NADH, FAD Lipogments and porphyrins, presented in brain tissues, relying on several contrasts: (1) Two-Photon Fluorescence (TPF) imaging, (2) SHG imaging, (3) fluorescence lifetime analysis and (4) Spectral analysis. This multimodality will offer more reliability and will increase the efficaicity of this endomicroscope.

In parallel to the instrumental endomicroscope development, we are building a large tissue database, that will be integrated in the endomicroscope. These specific multimodal signatures will help obtain robust optical discrimination factors between non-tumoral and tumoral tissues. In our previous works, we have demonstrated the reliability of TPF + SHG imaging technique for brain tissue diagnosis establishment. The proof of concept was validated by expert neuropathologists on different types of human brain tumor tissues where TPF + SHG images of our tissue database were compared and correlated with their corresponding H&E histological images^22^. In addition, and in the context of our database and using NIR and DUV excitation, we managed to discriminate quantitatively and qualitatively non-tumoral control brain tissue obtained in epilepsy surgery patients from primary and secondary tumors^22,28^. We managed also to discriminate low and high grade glioma^26^ through tracking molecular changes in both grades. Concerning meningioma, we have conducted a previous study to discriminate grade I and grade II meningioma qualitatively through TPF imaging and visbile spectroscopy^11^.

In this work, we aim to complete this preliminary study and to apply our new quantitative advanced analysis methods to robustly discriminate grade I and II meningioma tissues from control ones. For that purpose, we added the deep UV excitation range to combine its results with visible and NIR ones. We have multiplied the discriminants by focusing on the collagen variations observed in different grades, adding molecular ratios, indicators and thresholds and using advanced analysis method for spectral and lifetime results. Full field imaging and spectral analysis were conducted using deep UV excitation range to study the fluorescence response of tryptophan and collagen crosslinks in order to relate its fluorescence emission with its SHG emission using NIR excitation. In addition, Phasor approach were applied to investigate the fluorescence lifetime of NADH and FAD and to track their binding state changes.

## Results

### Spectral analysis

**Figure 1:**
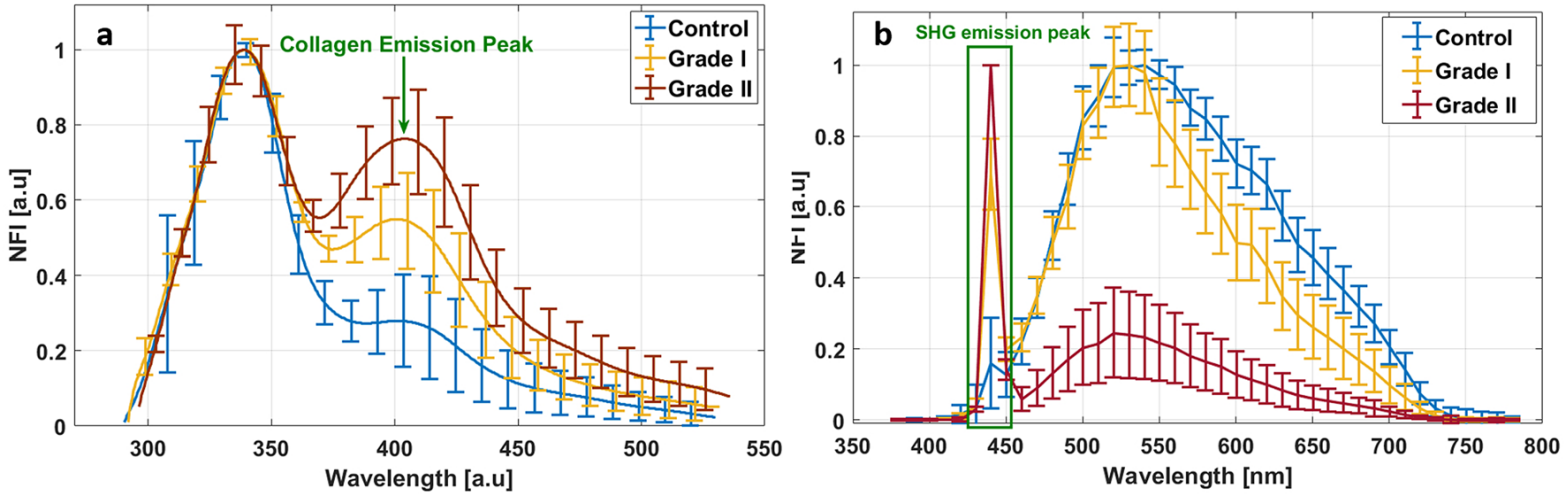
Spectral analysis using deep UV and NIR excitation range. (**a**) Normalized mean fluorescence intensity (NFI) spectra of 10 control, 7 grade I and 8 grade II meningioma samples acquired at 275 nm excitation. (**b**) Normalized mean fluorescence intensity (NFI) spectra of 24 control, 14 grade I and 11 grade II meningioma samples acquired at 890 nm excitation.

Using 275 nm as excitation wavelength, the autofluorescence of three endogenous fluorophores was highlighted: Tyrosine, Tryptophan and Collagen crosslinks, emitting respectively around 315 nm, 340 nm and 410 nm^28^. Normalized mean fluorescence intensity (NFI) curves were plotted to observe spectral shape variation between control (n = 10), grade I (n = 7) and grade II (n = 8) meningioma samples. For each spectrum we can observe two main peaks (Fig. 1a): The Tryptophan emission dominant peak around 340 nm and a secondary peak around 410 nm nm corresponding to collagen crosslinks emission. Control samples presented the lowest fluorescence emission of collagen crosslinks, while this emission increased in grade I and achieved a strong intensity level in grade II meningioma.

In NIR excitation range, spectral measurements were performed to highlight the TPF of NADH, FAD Lipopigments and porphyrins using 810 nm as excitation wavelength, and to underline the SHG emission of collagen structures using 890 nm as excitation wavelength. Figure 1b show the NFI curves using 890 nm as excitation wavelength for control (n = 24), grade I (n = 14) and grade II (n = 11) meningioma tissues. Observing the plotted spectra extracted from spectral images, a remarquable variation of SHG emission peak around 445 nm is noticed. This peak is very weak in control spectrum while it increases strongly in grade I mean spectrum. For grade II samples, it was noticed that the mean spectrum strongly lower that grade I and control samples and dominated by the SHG signal presenting a higher emission SHG peak. Indeed, fluorescence signal on these tissue types (detection range from 460 to 740 nm) shows also an important variation. The control tissues present a wider mean spectrum covering all the detection range, while grade I mean spectrum is narrower, with an intensity starting to decrease from 550 nm. This decrease corresponds to a lower emission of lipopigments, porphyrins I and porphyrins II, emitting respectively around 580 nm, 620 nm and 680 nm, in grade I compared to control samples. This behavior was also more noticed within grade II mean spectrum with no visible peak of lipopigments or porphyrins, indicating though a decrease of the emission of these fluorophores with the tissue grade. In addition, and since the obtained spectra reveal that collagen emission signal undergoes a remarquable variation with tumor grade, we decided to track the molecular changes of this macromolecule and to focus our discrimination study on it in order to extract more parameters based on its two types of emission signals and its concentration in tumors samples.

**Figure 2:**
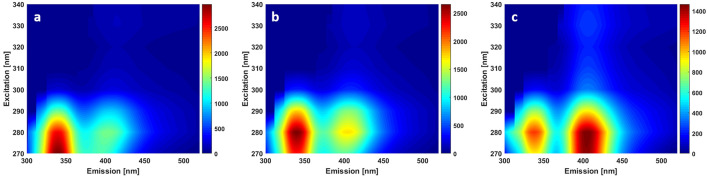
(**a**) Excitation Emission Matrix map in DUV excitation range for control (**a**), grade I (**b**) and grade II meningioma (**c**). The colorbars of each map reflects the emission intensity ot the spectral map.

In the DUV range, Excitation-Emission Matrix (EEM) were recorded to track the evolution of the spectral emission of collagen crosslinks. Starting from 270 nm, with 10 nm step, toward 340 nm, one sample was chosen from each tissue type to perform EEM measurements. EEM map for control, grade I and grade II meningioma are plotted respectively in Fig. 2a–c. These maps show that 270–280 nm is the most efficient range to excite tryptophan and collagen simultaneously. Each map presents two main emission zones, the first one around 340 nm corresponding to tryptophan emission, and the second one around 410 nm corresponding to collagen emission. These maps show clearly the increasing behavior of collagen fluorescence with the tissue grade, confirming the hypothesis concluded form NIR and DUV normalized spectra.

**Figure 3:**
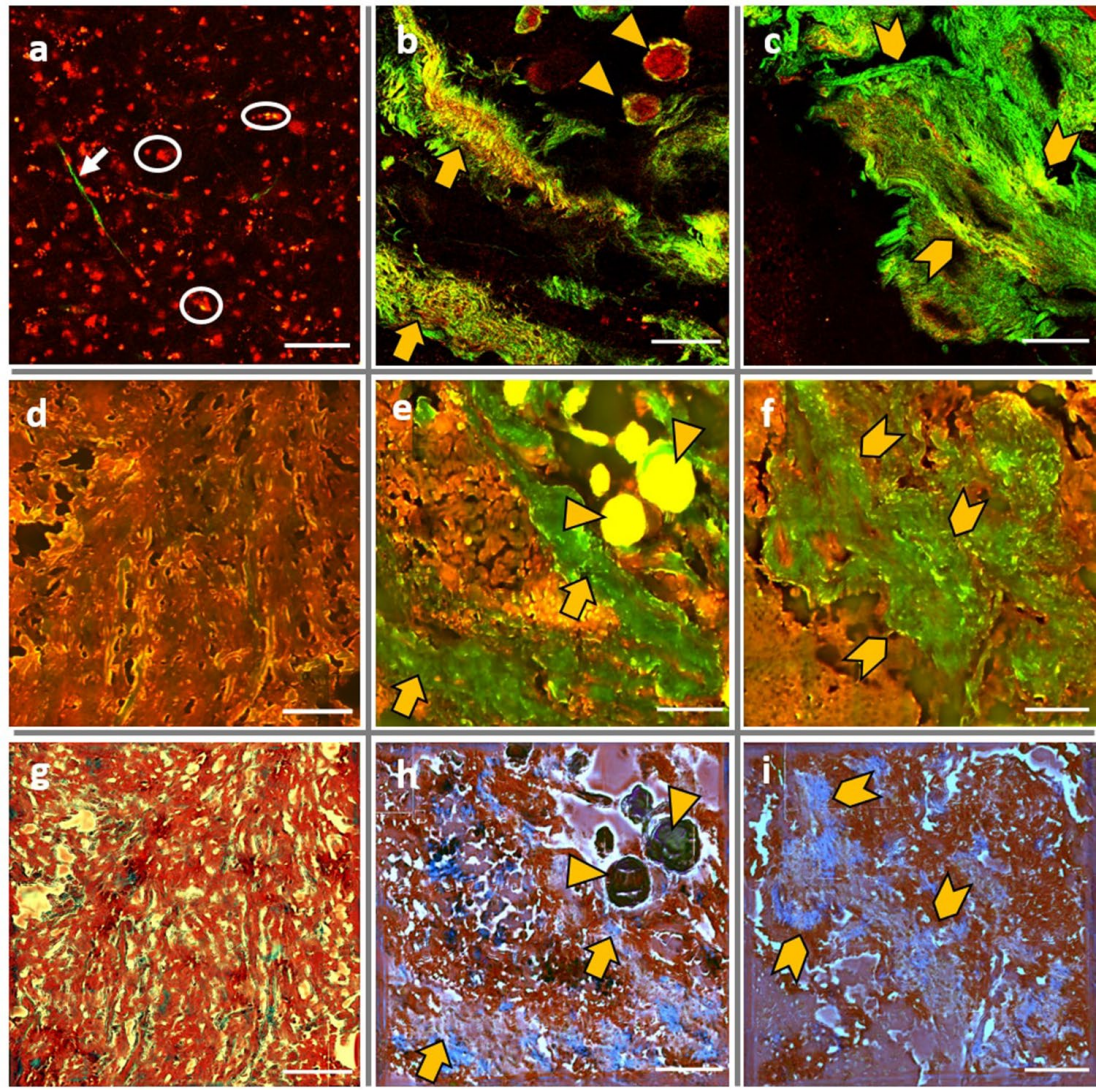
Image comparison through the NIR and DUV imaging set-up presenting a comparison between control (**a**,**d**,**g**), Grade I meningioma (**b**,**e**,**h**) and Grade II meningioma (**c**,**f**,**i**) through NIR confocal TPF + SHG images (**a**–**c**) 890 nm as excitation wavelength, and through DUV Tryptophan + Collagen full field images (**d**–**f**) using 275 nm as excitation wavelength. MT3 virtual staining Images (**g**–**i**); scale bar: 100 µm.

To complete our quantitative analysis and to illustrate spectral results, imaging acquisitions were performed on all samples using two different imaging setups. TPF (in red) and SHG (in green) images were acquired using 890 nm as excitation wavelength by merging the images of the two detection channels (Fig. 3a–c). Control TPF + SHG images (Fig. 3a) are dominated by a strong fluorescence signal and confirms the absence of collagen structures. This fluorescence is recognized by numerous red spots (white circles) identifying cells nuclei and sometimes overlapping some thin micro-vessels (white arrow). TPF + SHG of grade I meningioma (Fig. 3b) show the classical features of different grade I meningioma types where tumor cells forming fascicle whorls (orange triangles) with various amount of intercellular collagen structures as well as the presence of structured fibroblastic collagen and blood vessels with a thick wall (orange arrow). Grade II TPF + SHG images present a very dense collagen fibers network (orange arrows) with a blocky perivascular collagen structures, leading though to a stronger SHG emission dominating the fluorescence signal. These observations matches with the H&E staining meningioma images presented in our previous works, where TPF + SHG images were compared to their corresponding H&E images^11,29^.

In DUV excitation range, full field images of all tissue types were acquired using 275 nm as excitation wavelength. Images acquired through the tryptophan emission band-pass filter (307–223 nm) and through the collagen emission band-pass filter (408–438 nm) were merged and presented in Fig. 3d–f. Similar structures observed in the NIR images were also observed in DUV full-field images. These images present a qualitative and visual translation of the 275 nm spectral results (Fig. 1a) where control image are dominated by tryptophan emission (in red) with a low collagen intensity. For grade I and grade II images, the structures highlighted by SHG in the NIR images can be seen in DUV images (in green). The last row consists of the virtual staining images generated from DUV full field images using Masson’s Trichrome (MT3) virtual staining method^30^. The generated images of control, grade I and grade II meningioma (Fig. 3g–i respectively) were well matched to their equivalent DUV images noticing that collagen zones were clearly stained in blue and differentiated from the other images structures.

**Figure 4:**
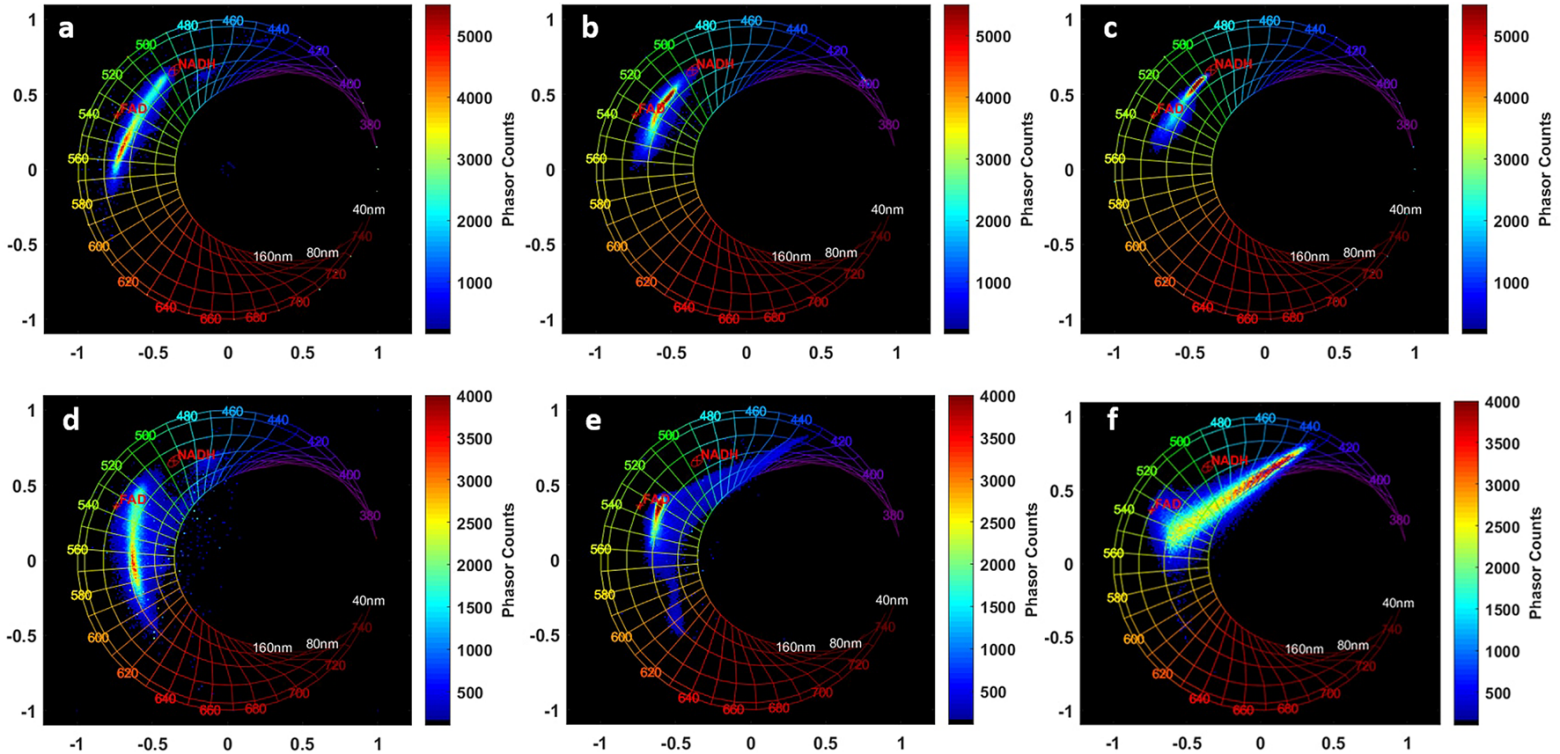
Spectral Phasor histograms using 810 nm (**a**–**c**) and 890 nm (**d**–**f**) for: Control (**a**,**d**), Grade I meningioma (**b**,**e**) and Grade II meningioma (**c**,**f**).

To better visualize spectral variations, and since we cannot excite NADH using 890 nm, spectral measurements were perfomed using 810 nm. The spectral phasor technique was used to analyze spectral images acquired under both 810 and 890 nm. This approach simplifies the analysis of the global emission spectra of each tissue type with an unmixed view of the spectral contributions of each molecule. Acquired at 810 nm, spectral phasor histograms of each tissue type were plotted in Fig. 4 for control (Fig. 4a), grade I meningioma (Fig. 4b) and grade II meningioma (Fig. 4c). In addition, fingerprint Phasors of NADH and FAD measured in standard solutions were added in red at 495 nm and 535 nm respectively for each histogram to better visualize the emission of each molecule in each tissue type phasor histogram.

From control tissues to higher grades, we observed that the emission cloud undergoes a shift from lipopigemnts esmission zone towards towards NADH fingerprint. In control phasor histogram the emission cloud is focused around the FAD-lipopigments emission zone around 540–560 nm. In grade I phasor histogram, the cloud is focused inside the FAD and NADH fingerprint showing a strong drecrease of lipopigments emission, while in grade II phasor histogram the emission peak cloud continues its crawling toward the NADH fingerprint, pointing a variation in the concentration of these molecules when going from a grade to another. The same analysis is shown in Fig. 4d–f but for 890 nm excitation wavelength. At this wavelength we are able to detect and highlight the SHG emission of collagen structures. The same trend is observed for 810 nm and 890 nm phasor histograms. In control samples, the emission cloud is strongly shifted toward the FAD and lipopigments emission zone without detecting a strong SHG emission. In grade I meningioma histogram, the emission is also focused near the FAD fingerprint with a shift tail toward the SHG emission zone, while the grade II meningioma emission cloud is strongly shifted toward the SHG zone due to a strong emission of this signal.

### Molecular ratios

**Figure 5:**
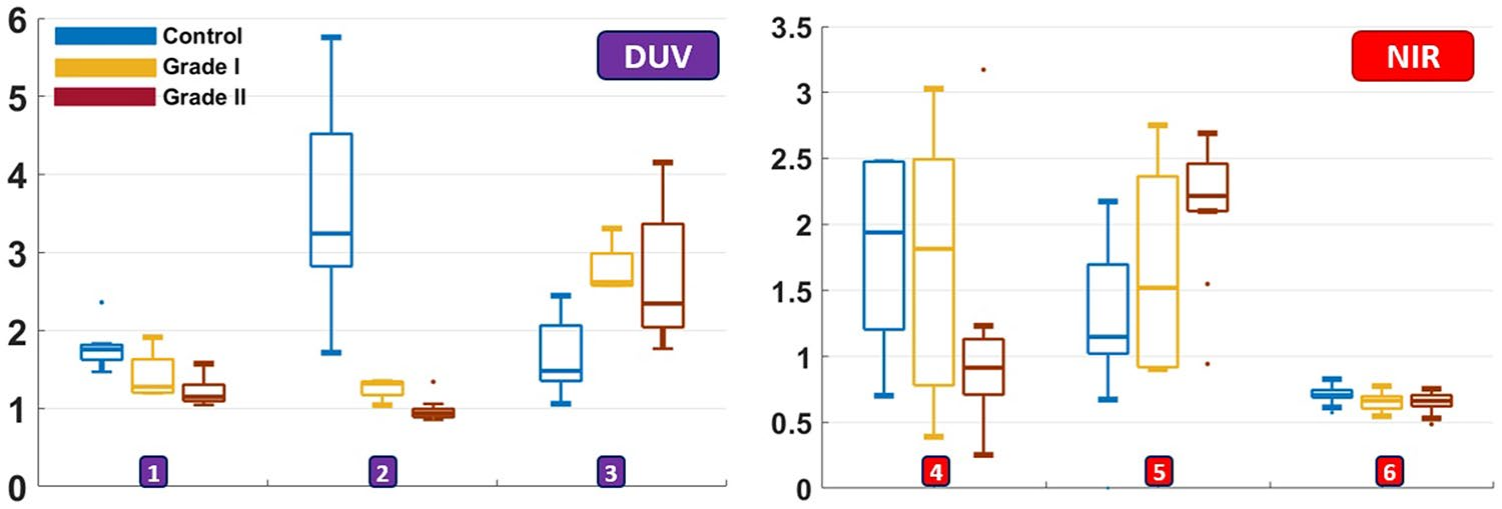
Group boxplots of six molecular ratios acquired from spectral data at 275 nm (DUV) and 810 nm excitation (NIR) for control, grade I and grade II meningioma. DUV part shows Tryptophan/Collagen (N1), Tryptophan/NADH (N2) and Tryptophan/Tyrosine (N3) ratios. NIR part shows PN ratio (Porphyrins/NADH) (N4), ratio LP (Porphyrins/Lipopigments + Porphyrins) (N5) and redox ratio (FAD/ (NADH + FAD)) (N6) ratios.

To complete molecular emission tracking, several molecular ratios related to the excited molecules were compared through 275 nm and 810 nm spectral measurements. Figure 5a gathers the molecular ratios extracted from DUV spectral data using 275 nm excitation. Three molecular ratios for control, grade I and grade II meningioma are displayed. Tryptophan/Collagen ratio (Fig. 5a N1), Tryptophan/NADH ratio (Fig. 5a N2), and Tryptophan/Tyrosine ratio (Fig. 5a N3). As expected, a decrease in the Tryptophan/Collagen ratio from Control to grade I and grade II meningioma discriminates significantly these three tissue types. The Tryptophan/NADH ratio was clearly higher in control tissues and discriminates significantly these three tissue types in a descending behavior with tumor grade. This descending behavior indicates that the NADH level increase with the tumor grade to reach a higher presence in grade II meningioma. This results are weakly detectable on the EEM map of grade II meningioma (Fig. 2c), where at 340 nm excitation, a faint emission tail around 450 nm appears close to the collagen emission zone. This tail is very low in control EEM map, and slightly present in grade II EEM map, confirming the observed behavior in Tryptophan/NADH ratio.

As for DUV, exploitation of the three molecular ratios extracted from NIR spectral data using 810 nm excitation wavelength reveals and confirms another molecular variation behavior with the tumor grade; PN ratio (Porphyrins/NADH), LP ratio (Porphyrins/Lipopigments + Porphyrins) and redox ratio (FAD/(NADH + FAD)) are displayed in Fig. 5b (N3, 4 and 5 respectively). The PN ratio decreases from control to grade II, revealing a loss in porphyrins contribution in the meningioma samples. Similarly, the LP ratio reveals a decrease of the lipopigments contribution in grade I and grade II meningioma, translated by an increasing variation of the ratio from control to grade II. This molecular variation in term of emission contribution of lipopigments and porphyrins confirms the changes in the mean normalized spectra acquired at 890 nm (which is better than 810 nm to excite these two molecules) of the three tissue types in the emission range of the cited molecules.

### Collagen 3D discrimination

**Figure 6:**
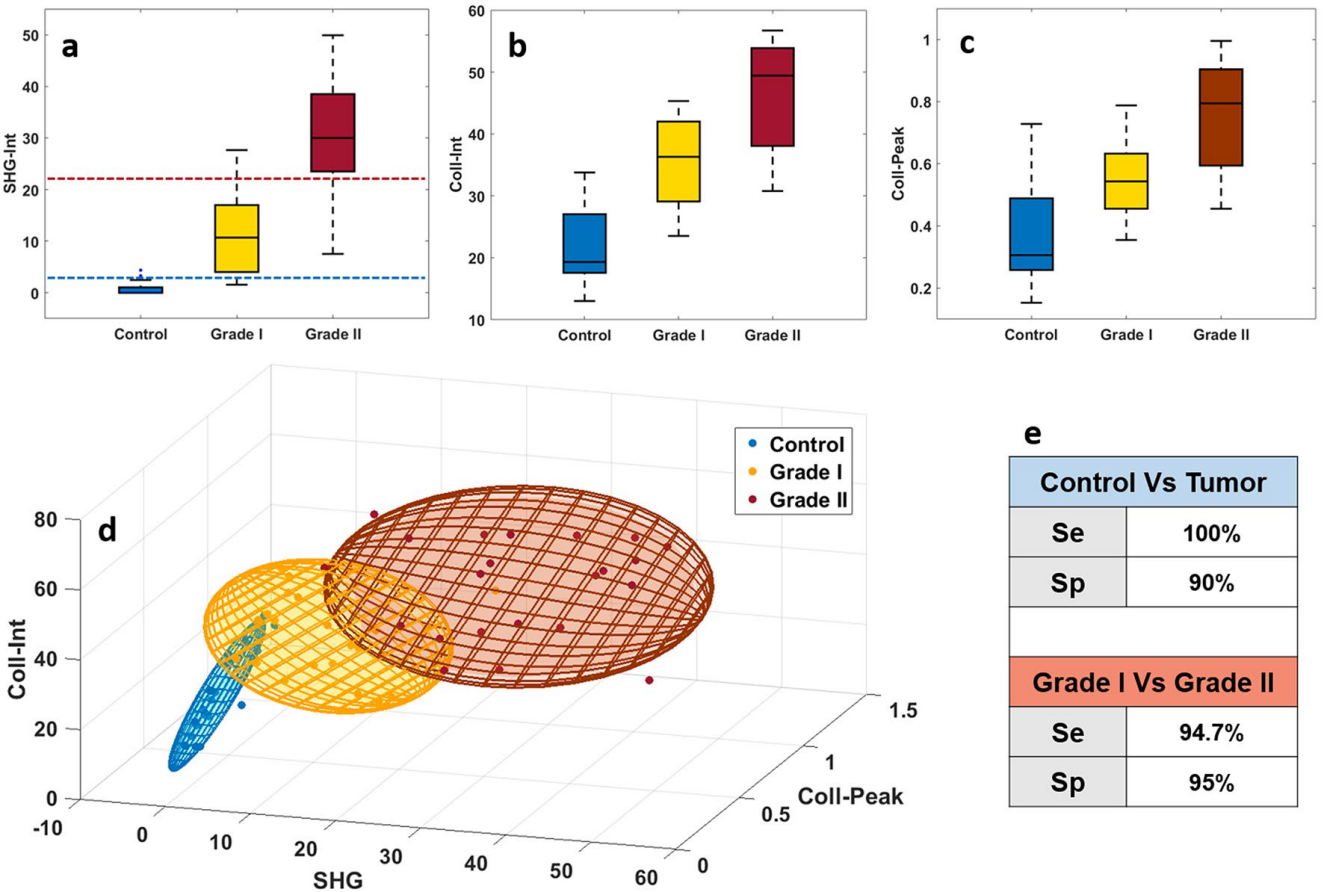
(**a**) Boxplot view of SHG integral proportion (SHG-int) in the total spectra at 890 nm excitation wavelength for control, grade I and grade II meningioma; (**b**) collagen maximum intensity peak (Coll-peak) using 275 nm as excitation wavelength for control, grade I and grade II meningioma; (**c**) Collagen integral proportion (Coll-int) in the total spectra acquired using 275 nm Control, Grade I and Grade II meningioma. (**d**) 3D-discrimination ellipsoids of control, grade I and grade II meningioma where SHG-int, Coll-peak and Coll-int were taken as 3D coordinates. (**e**) table gathering the sensitivity and specificity of discrimination test results realized for 10 control vs. 15 tumor samples and for 7 grade I vs. 8 grade II meningioma samples.

Based on the previous observation, all results concerning the collagen emission tracking in NIR and DUV were grouped to build a discriminative algorithm between control-tumor and between grade I-grade II meningioma. First, a boxplot representing the SHG integral proportion (SHG-Int) in the total emission spectr at 890 nm of each tissue type was plotted in Fig. 6a. We observed, the same trend than normalized plotted spectra and spectral phasor histograms in NIR excitation domain, with a significant increase of the SHG emission observed from control samples to grade II meningioma. Secondly, boxplots plotted in Fig. 6b,c show the integral proportion of collagen emission (coll-int) in the total emission spectra and the normalized maximal emission collagen peak (coll-peak) respectively, acquired from 275 nm spectral results in the DUV excitation domain. These two boxplots highlight the increase of the collagen emission with tumor grade, from a low contribution on control samples to a higher emission in grade II meningioma.

Based on the obtained differentiation, a homemade 3D discrimination algorithm, used in previous works^26^, was applied to these set of data.This 3D algorithm combines three different parameters extracted from each tissue type in order to plot a 3D scatter cloud which is the approximated by a Gaussian ellipsoid using the mean and the standard deviation as parameters for the covariance of the ellipse covering 60% of the total probability mass.

As threshold, we choose SHG-Int to establish a Control-tumor diagnosis response (Fig. 6d) and a grade I–grade II discrimination. For the coordinates, we choose the SHG emission as X axis parameter, Coll-Peak as Y axis parameter and Coll-int as Z axis parameter.

Two discrimination tests were performed, the first one is a control-tumor diagnosis using the 2.5 value of SHG-Int as threshold. Applied on the sets of data, we assumed in this test that if the point is inside a grade I or grade II ellipsoid and its SHG-Int is higher than 2.5, tumor can be diagnosed and confirmed. With 0% of overlapping volume between control and tumor ellipsoids this test reaches a 100% sensitivity and a 90% specificity. The second test was a grade I-grade II meningioma discrimination using the 22 value of SHG-Int as discrimination threshold. Applied only on grade I and grade II sets of data, we assumed that if the point is inside the grade I ellipsoid and its SHG-Int is lower or equal than 22, grade I is detected; while if the point is inside the grade II ellipsoid and present a SHG-Int value higher than 22, grade II is detected and confirmed. This test reaches a 94.7% sensitivity and 95% specificity.

### Fluorescence lifetime analysis

**Figure 7:**
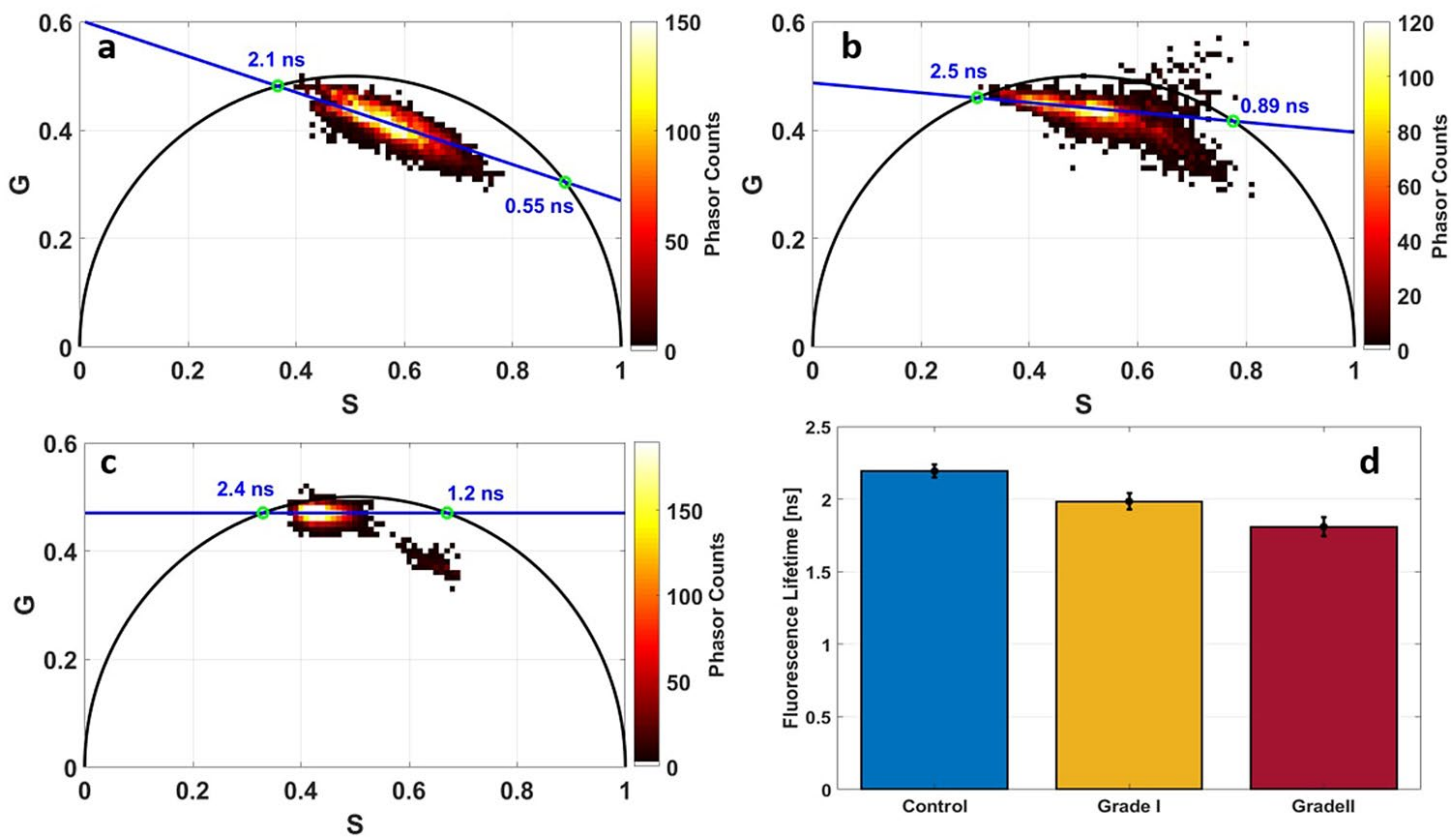
Fluorescence lifetime analysis of FAD (**a**–**c**) and NADH (**d**). Phasor FLIM histogram of FAD using 810 nm as excitation wavelength for control (**a**), grade I meningioma (**b**) and grade II meningioma (**c**), free and protein-bound FAD lifetime values are drawn as green circles intersecting with the universal circle; mean lifetime histogram of protein-bound NADH at 810 nm for control, grade I and grade II meningioma.

Figure 7a–c shows respectively the phasor Flurerescence Lifetime Imaging (FLIM) histograms for FAD using 810 nm for the three tissue types: control, grade I and grade II meningioma. Different lifetime values of the two FAD components were obtained: control samples had the lower free FAD lifetime (2.1 ns) while grade I and grade II had higher values (2.5 and 2.4 respectively). The same trend was obtained for protein-bound FAD lifetime, where control samples presents the lowest value (0.55 ns) and the grade I and grade II had higher values (0.89 and 1.2 respectively).

For NADH lifetime, we used a bi-exponential fit to extract the fluorescence lifetime of its two components. Since that Protein-bound NADH lifetime can discriminate non-tumoral control brain tissue obtained in epilepsy surgery patients from low and high grade glioma^26^, histograms representing the lifetime of this component were plotted in Fig. 7d. It is clear that the lifetime of protein-bound NADH decreases with the tumor grade, and can discriminate significantly control from tumors, and grade I from grade II meningioma. A mean value of 2.19 ± 0.046 ns was found for control, 1.98 ± 0.055 ns for grade I and 1.8 ± 0.065 ns for grade II meningioma.

**Figure 8:**
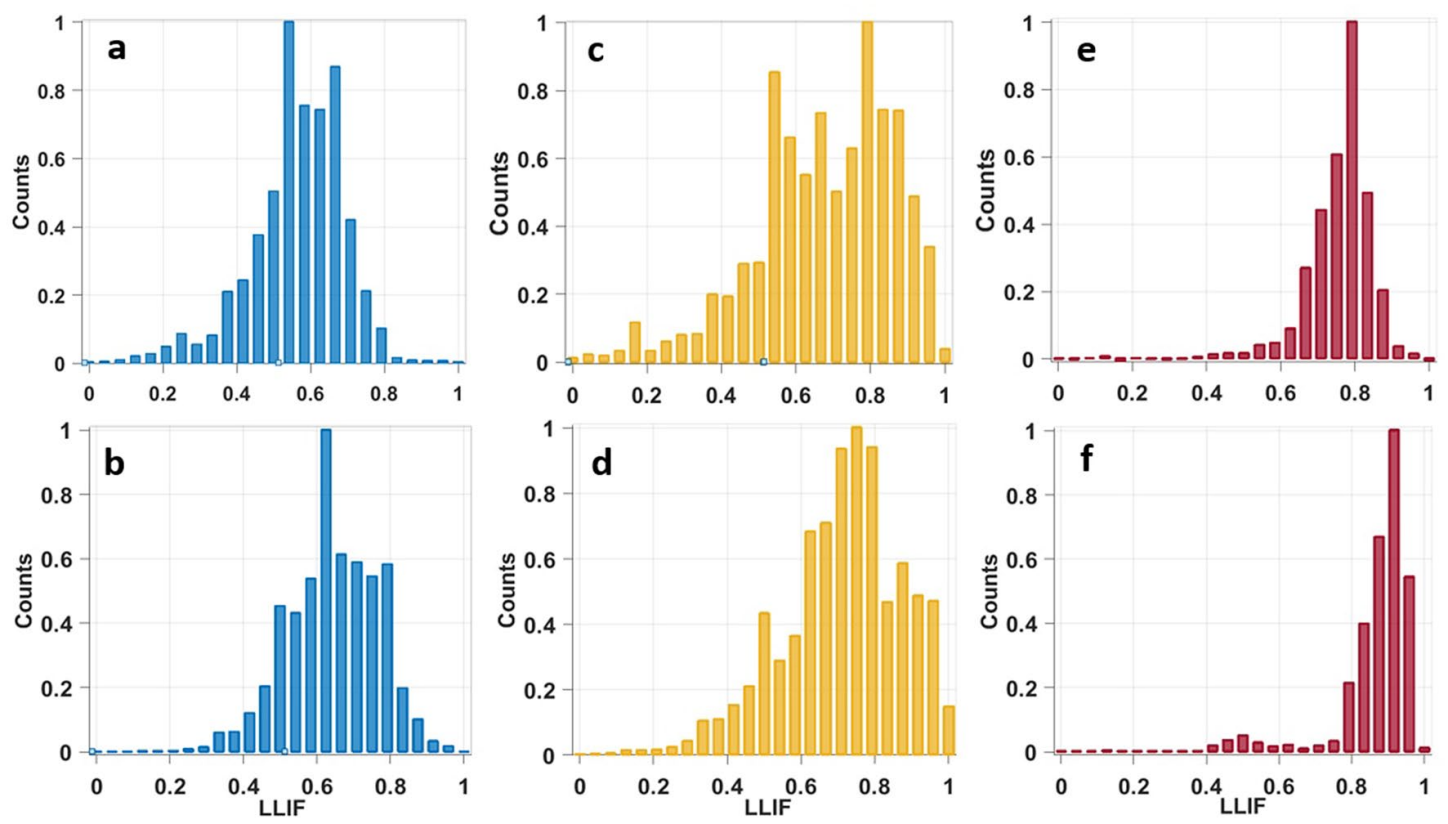
Long lifetime intensity fraction (LLIF) histogram of NADH (**a**,**c**,**e**) and FAD (**b**,**d**,**f**) issued from phasor FLIM analysis at 810 nm for Control (**a**,**b**), grade I meningioma (**c**,**d**) and grade II meningioma (**e**,**f**).

In addition to lifetime values extraction, we tracked the variation of the contribution of each molecule component in each tissue type through their FLIM phasor histograms. For that purpose, we regrouped all FAD and all NADH FLIM images from all types, in order to build a global phasor histogram for each molecule. Then these global phasor histograms were used to plot the histogram of Long Lifetime Intensity Fraction (LLIF) distribution of each molecule component in each tissue type (see data analysis). This parameter helps us to determine the fraction of fluorescence emitted by each molecule component presented in a FLIM image in each tissue type. LLIF histogram of NADH (Fig. 8a,c,e) and FAD (Fig. 8b,d,f) are shown respectively in Fig. 8 for control (a,b), grade I (c,d) and grade II meningioma samples (e,f).

Observing the plotted histograms, we can clearly notice that the distribution of LLIF of these two molecules present the same variation behavior from grade II to control and undergoes a shift from high LLIF values toward lower values. For grade II meningioma, Fig. 8e,f show a strong accumulation of higher LLIF values with a narrow distribution. While in grade I, the histogram undergoes a shift toward lower LLIF values with a wider distribution than grade II including high and lower LLIF values in Fig. 8c,d. In control samples, the histogram shifts even more toward lower LLIF values presenting a decrease of the counts in high LLIF values region (Fig. 8e,f).

## Discussion

To complement a past study^11^, we aimed in this work to analyze and to track changes of brain tissue auto-fluorescence at the molecular level through advanced multimodal quantitative analysis; and to correlate those changes with meningioma tumor grade evolution. Our goal is to establish several discrimination parameters between control, grade I and grade II meningioma tissues. We used multiscale excitation, from DUV to NIR, and different imaging instruments to investigate the fluorescence emission of several endogenous fluorophores on a large cohort of samples. Advanced analysis was performed on the results of those various imaging modalities, they consisted in analyzing and correlating the spectral and time-resolved variations of the auto-fluorescence response of the three tissue types.

At first, we investigated the spectral response changes of meningioma grades, in DUV range under 275 nm excitation, of two major molecules: tryptophan and collagen crosslinks. Tryptophan residues have been proposed as a fluorescent marker in the study of phosphorylation of cellular mechanisms that regulate cell growth, proliferation and transformation^27,28^. Therefore, many studies relied on the auto-fluorescence signal of this molecule and its related metabolic ratios to discriminate cancer cells^27,28^. While collagen plays multiple roles in the composition of the different tissue compartments of the central nervous system^31^. Indeed, Type VI collagen can be found in connective tissues, mainly consisting of central nervous system basal lamina associated with the meninges and the brain blood vessels and it’s considered as a good marker for tissue vascularization^31^.

The spectral results acquired in DUV and in NIR range (Figs. 1, 2, 4d–f) shows a significant increase of collagen fluorescence and SHG emission when passing from control to gigher grades. In fact, grade II meningioma tissues feature prominent blocky perivascular and interstitial collagen (Fig. 3c), which occasionally coalesces into large acellular zones or forms brightly eosinophilic amianthoid-like collagen^4^. These coalescing blocks increase the ability of such structures to generate a non-linear signal such as SHG. These blocks hide the tumor cells zones and decrease their capacity to absorb light, explaining the strong and dominant SHG signal emission and the weak fluorescence response in grade II meningioma samples (Fig. 1b). On the other side, grade I meningioma features (Fig. 3b) spindled tumor cells forming parallel and interlacing bundles in a various amount of intercellular collagen rich matrix^4^. This fact could explain the presence of a high fluorescence signal in grade I samples with a low emission (comparing to grade II) of SHG signal due to a more homogenous repartition of collagen matrix in these samples.

In our previous proof of concept work, TPF + SHG images of were compared to their corresponding H&E stained images in order to validate the efficiency of two-photon microscopy to reveal the necessary information for a reliable diagnosis information on tissue’s nature^22^. Therefore, and since H&E staining is time consuming and involves laborious preparation, we tried in this study to attempt the use of virtual staining of microscopy images through deep learning to compare its resulting images with our DUV full-field images. In their recent work, Rivenson et al.^30^ shows that a convolutional neural network trained using a generative adversarial-network model can transform wide-field auto-fluorescence images of unlabeled tissue sections into images that are equivalent to the bright-field images of histologically stained versions of the same samples. Realized on a large cohort of liver and lung tissue samples, the staining efficiency of their approach, was blindly evaluated by a group of pathologists who were able to recognize histopathological features in their output images, and achieved a high level of agreement with the H&E stained images^30^. In addition, this approach can be applied to other microscopy modalities that use other endogenous or exogenous contrast markers^30^. So as a first step, we decided to test this approach on our full-field images generated using 275 nm in order to explore its ability to recognize same features as H&E stained images of brain samples. The obtained images were encouraging in term of collagen network staining, but the staining of isolated cells was not sufficiently effective. This test opens the door to realize a large study using this virtual staining method and impose the training of this approach on the corresponding H&E stained brain images of our full-field images and the use of large cohort of sample images of different tissue types, before applying it on confocal NIR images.

Concerning metabolic ratio indicators, the extracted ratios translated the same variations of collagen concentration observed by spectral analysis. In addition, it was noticed from Tryptophan/NADH ratio (Fig. 5a N2) and from the EEM map of meningioma samples that an increase of NADH levels appears in grade II meningioma compared to control samples.

But since the absorption cross section of NADH is low in the DUV^32^, spectral measurements were acquired using two-photon excitation in NIR at 810 nm to efficiently excite this molecule and to better track its variation with tissue type. Through spectral phasors at 810 nm (Fig. 4a–c), the increase of NADH level with tissue grade was confirmed and clearly noticed. This increasing behavior of NADH levels in tumor tissues was reported in several studies in literature and observed in rat brain tissues^33^, human fixed tissues^34^ and in tumoral esophageal epithelium cells^35^. In fact, NADH is the reduced form of Nicotinamide Adenine Dinucleotide (NAD) which is a coenzyme that is central to metabolism and found ubiquitly in all living cells^32^. This molecule is involved in cell metabolism processes as an electron carrier in redox reactions^32^. It had been widely monitored to evaluate oxidative metabolic state in cells and tissues^36^ and considered as the most prevalent endogenous molecule. Our previous studies on glioma^26^ and other literature studies^33^ proved that the observation of higher value of NADH in a tissue indicates a higher malignancy. In meningioma tumors, the mitotic activity and cellular proliferation increase in proportion with the grade^4^, leading to an increase of metabolic activity with the grade, explaining the increase of NADH level in proportion to tissue grade, reaching a high level of NADH in grade II meningioma.

Another molecular change in meningioma grades was noticed through NIR spectral results cooresponding to a variation of lipopigments and porphyrins levels in tumor tissues, where the fluorescence signal of these two molecules was more important in control tissues and decrease in tumor samples. In literature, lipid levels in brain tissues were found decreasing with tumor grade, this decrease is linked to the sensitivity of lipopigments to the metabolic state of the tissue even if it is not tumoral and that they are affected by several metabolic processes such as oxidative and glycolytic metabolism^34,37^. Studies in the literature have shown a spectral distortion and a strong decrease of lipopigments level in fresh tissues five hours after resection due to the metabolism alteration after the resection and the end of the processes cited above^34^.

In the light of the obtained results of collagen variation in the DUV and NIR range, we decided to use a homemade 3D discriminative algorithm, already tested in a previous work^26^ with DUV molecular ratios. These excellent obtained percentages of sensitivity and specificity for control vs tumor (100% sensitivity and 90% specificity) and grade I vs grade II discrimination (94.7% sensitivity and 95% specificity) constitute a strong encouragement to train more this algorithm with a larger number of samples to confirm its robustness. It will constitute an important tool that will serve for future blind analysis in order to acquire fast diagnosis on tissues nature and its grade.

Moving to time-resolved fluorescence results, protein-bound NADH lifetime were extracted from decay curves of different region of interest (ROI) in FLIM images through a bi-exponential fit process. This component presents the majority of NADH presence in human cells^35^. In control samples, this component lifetime was found high and decreased with the tumor grade. The same variation was obtained in our past study using 810 nm as excitation wavelength^11^; where NADH lifetime value was higher in grade I than grade II (2.67 ± 0.15 for grade I and 2.55 ± 0.14 for grade II at 810 nm; and 4.00 ± 0.18 for grade I and 3.64 ± 0.10 for grade II at 405 nm^11^. The difference between the two works is due to the fact that mono-exponential fit was used to fit the decay curves in the previous work, while in the present one, we used bi-exponential fitting to calculate precisely the protein-bound NADH lifetime.

The bound-NADH lifetime values obtained in this study were significantly different between control-tumor discrimination (student test: *p* value = 0.00013 for control vs grade I) or between grade I–grade II discrimination (student test: *p* value = 0.0077 for grade I vs grade II). The increase of fluorescence lifetime can be correlated with an increase of NADH concentration in tumor tissues, confirmed through 810 nm spectral analysis, and considered as a reliable discrimination threshold criterion in a diagnosis rapid test. So a high NADH level with a lifetime value lower than 1.9 ns leads to a grade II meningioma diagnosis; a low level of NADH and a lifetime value higher than 2.1 ns leads to a non-tumoral control brain tissue obtained in epilepsy surgery patients zone diagnosed while if these two criteria were in between, a grade I could be diagnosed.

Phasor FLIM technique was also used to analyze FLIM data acquired at 810 nm to study FAD and its two components, free and protein-bound, lifetime values in the NIR domain where lifetime values varies in each type as well the phasor cloud shape. This variation in the phasor cloud shape led us to conclude that the repartition of the two FAD components seems to be changing from one type to another. Therefore, we investigated the fluctuations of the two components of FAD and NADH, by observing their Long Lifetime Intensity Fraction (LLIF) histograms, in order to determine the variation in term of the contribution of each component in each tissue type.

Therefore, it was remarquable that the LLIF histograms of these two molecules follow a similar trend from control to higher grades. Free FAD, which corresponds to higher LLIF values, was more present in grade II meningioma than protein-bound FAD (lower LLIF values), inducing a strong shift of the histogram toward the right side (Fig. 8e,f). In grade I meningioma this strong shift is not present while the histogram is wider (Fig. 8d) shifted toward lower LLIF values, therefore free FAD is more concentrated in grade II than grade I. Control samples present a histogram shape (Fig. 8f) similar to grade I but with a lower accumulation of high LLIF values and an increase of low LLIF values, which means a higher protein-bound FAD occurrence and a decrease of free FAD concentration.

Similar shift and variation in protein-bound and free components presence is observed for NADH histograms (Fig. 8a, c, e). Protein-bound NADH is less present control samples, its level increase in grade I samples, while it was strongly presented in grade II samples with a strong decrease of free NADH levels. These observations allow to conclude that NADH and FAD LLIF repartition shift to higher values with the tumor grade. We can assume and confirm, through our two works on low and high grade glioma^26^ and through this work on grade I and grade II meningioma, that the concentration NADH and FAD components follow a variation behavior with the tumor grade and with the malignancy of the tissue and can become a good discriminative indicator between tumor grades.

## Conclusion

We presented in this study an advanced multimodal approaches to analyze and track the molecular variation between control, grade I and grade II meningioma tumor. We can conclude at the end this work, that the tracking of several molecules individually (collagen, tryptophan, Lipopigments, NADH, FAD) and their analysis through multiscale and multimodal analysis could lead to better extraction of discriminative parameters tissue differeciantion.

The combination of these parameters with qualitative analysis allows reaching our to increase the reliability of the future tissue differenciation based on this database and to render our tissue database more robust.

The molecular changes and the significant discrimination quantitative factors between control and meningioma tumor tissues that were extracted in this study could be enumerated as:

1. increase of collagen fluorescence signal with the tissue grade: a tissue with a collagen contribution (Coll-Int) lower than 28 is considered as control, while a tissue with a Coll-Int higher than 28 is considered as tumor.
2. increase of SHG contribution with the tissue grade: a tissue with a SHG contribution (SHG-Int) less than 2.5 is diagnosed as control, between 2.5 and 22 is diagnosed as grade I, and higher than 22 is diagnosed as grade II meningioma.
3. increase of protein-bound NADH concentration with the tissue grade with a decrease of this NADH component lifetime: a tissue with a bound-NADH lifetime higher than 2.1 ns is diagnosed as control; between 1.9 and 2.1 ns is diagnosed as grade I, while lower than 1.9 is diagnosed as grade II.
4. increase of free FAD concentration with the tissue grade and an increase of this FAD component lifetime: a tissue with bound-FAD lifetime lower than 0.7 ns is diagnosed as control; between 0.7 and 1 ns is diagnosed as grade I, while higher than 1 ns is diagnosed as grade II.

However, the obtained results should be strengthened with more statistics by increasing the samples number. In addition, while control samples group of this study consisted of brain parenchyma tissues extracted through epileptic surgery, a second control group should be added in the future that consist of healthy dural matter since that residual meningioma tissues are usually found in the surrounding meninges.

The clinical relevance of such multimodal optical analysis, which can be easily applied to a neurosurgical purpose was confirmed. Each specific tissue signature, established in our database, will be implemented in the under-development endomicroscope to give it the ability to establish a reliable real-time diagnosis response.

More technical detailed information (pulse duration, pulse compression system, endoscopic fiber) about this endomicroscope characteristics are already published in previous works of our team^38,39^. In addition, a handheld miniature imaging probe head is under development and presented in a previous work^40^, and it will be coupled with this endomicroscope, to ensure in-vivo image acquisitions.

This endomicroscopic imaging probe head could be directly implemented in the human brain while performing its measurements. The rapidity of acquisition and diagnosis response establishment of this tool is an advantage over the actual used tools (extemporaneous examination, intra-MRI… etc.). Additionally, our tool does not require the use of any exogenous markers which simplify the surgery workflow and limits any biased classification.

However, this technical approach may suffer from several future limitations during its use intraoperatively. The blood presence in the surgical cavity, and due to its high absorption, may alter the fluorescence signal and consequently the image contrast, which imposes the elimination of such blood accumulation within the examined region.

Indeed, the imaging probe head should be fabricated with biocompatible materials and should Withstand repeated sterilization process which exposes the probe to high temperatures, which may affects the endoscopic fiber structure in the future.

## Materials and methods

### Samples

In cooperation with the neurosurgery and the neuropathology department of Sainte Anne hospital (Paris), 49 fresh samples from were acquired according to the approval of the Sainte-Anne Hospital – University Paris Descartes Review Board (CPP Ile de France 3, S.C.3227). All methods were carried out in accordance with the relevant guidelines and regulations of this approval and informed consents were obtained from all patients. For NIR measurements we used a samples cohort which consisted of 14 grade I meningioma, 11 grade II meningioma and 24 control samples obtained from epileptic surgeries, while for DUV measurements the number of samples were 10 control, 7 grade I and 8 grade II meningioma samples.

After the surgery, each sample was directly sent in a normal saline solution to the neuropathology department where our visible endoscope is located. After the acquisition of the spectral measurements, the sample was sent to the IMNC laboratory (Orsay-France) where our NIR excitation multimodal setup is located. After the NIR excitation measurements, these samples were stored at − 80 °C. For deep UV measurements, the samples were put at − 18 °C few hours before to be prepared to cutting, after that, and using a cryostat (CM 1950, Leica Microsystems), they were cut into 10 μm slices then fixed with ethanol (100 °C) and stored at 4 °C until experimentation. These fixed slices were then used for full field imaging and spectral measurements on the Deep UV setup at DISCO Beamline.

### Deep UV excitation setup

Deep UV spectral measurements were carried out on DISCO beamline^41^ at Synchrotron SOLEIL (Saint-Aubin, France). On this beamline, spectral and full-field imaging measurements were performed using 275 nm as excitation wavelength. The experimental setup consists of a full-field microscope (Zeiss Axio-observer Z-1) to acquire images and a micro-spectrofluorimeter (Olympus IX71) to perform spectral measurements.

For each sample, a large mosaic was selected to perform Tryptophan + Collagen full field imaging acquisitions. Two band-pass filters, (1) 323-357 nm and (2) 408-438 nm (Semrock, USA) were serially positioned in front of a CCD camera (Pixis BUV, Princeton Instrument, USA) to select respectively the fluorescence signal of tryptophan and collagen.

The obtained full field images were reconstructed and merged through an open source-image-processing software “FIJI”. Afterwards we used Masson’s trichrome (MT3) model to generate virtual stained images from the merged Tryptophan + collagen images. This model consists of a deep-learning-based virtual histology staining method of label-free human tissue samples. The network models for Masson’s trichrome (MT3) stain alongside sample test-image data are available through a Fiji-based plugin^30^. This plugin is publicly available and can be downloaded through DeepImageJ website in bundled models section: https://deepimagej.github.io/deepimagej/models.html.

For spectral measurements, several Regions Of Interest (ROI) in the acquired mosaic were then selected to perform spectral measurements through the micro-spectrofluorimeter. Each ROI have a size of 160 × 160 µm with 4 µ as pixel size. From each pixel, the collected spectrum is the sum of the fluorescence emission of four endogenous fluorophores: Tyrosine, tryptophan, collagen and NADH.

### Multimodal NIR excitation setup

This multimodal setup is able to perform four different optical imaging modalities: (1) TPF imaging, (2) two-photon spectral imaging, (3) SHG imaging and (4) two-photon Fluorescence Lifetime Imaging (FLIM) measurements. It consists a TCS SP8 MP confocal two-photon microscope (Leica Microsystems, Wetzlar, Germany) coupled with a FLIM module from PicoQuant (GmbH, Berlin, Germany) which uses Time Correlated Single Photon Counting (TCSPC) technique to carry out FLIM acquisition through its dedicated software (Symphotine, Picoquant, Germany). More details of this microscope are published elsewhere^11,26,39^.

The collected fluorescence signal is collected into two detection channels where in the first one (channel 1) a 448 nm centered band pass filter (FF01-448/20-25, Semrock, USA) was introduced, while in the second one a 520 nm centered band pass filter (FF01-520/35-25) was introduced.

The TPF + SHG images acquisition were performed using 890 nm as excitation wavelength. Using this wavelength, the channel 1 allows to select the SHG emission signal while the channel 2 allows to select the FAD emission signal. These images were acquired through the microscope dedicated Leica software (LAS-X) anf then reconstructed through the open source-image-processing software “FIJI”.

FLIM images acquisition were performed using 810 nm as excitation wavelength. Using this wavelength, the channel 1 allows to select the NADH emission signal instead of SHG, while the channel 2 selects always the FAD emission signal.

For spectral imaging acquisition, and instead of using channel 1 and channel 2, an internal hybrid detector was used. It covers a detection range from 380 to 790 nm with 10 nm as chosen spectral resolution.

### Data analysis

#### DUV spectral analysis

DUV Spectral data processing was conducted using a home-developed software in DISCO beamline at Soleil synchrotron, based on matlab data processing codes used in several previous works^28^.

**Figure 9:**
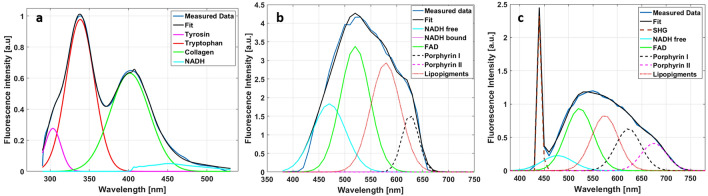
Examples of spectral fitting of DUV and NIR collected data. (**a**) Fitted spectrum acquired using 275 nm as excitation wavelength (**a**); (**b**) fitted spectrum acquired using 810 nm as excitation wavelength; (**c**) fitted spectrum acquired using 890 nm as excitation wavelength.

The excitation under 275 nm highlight the fluorescence emission of four fluorophores: tyrosine (tyr), tryptophan (tryp), collagen (coll) and NADH. For each collected spectra, a spectral fitting process was performed to extract the emission contribution of each fluorophore. This emission was represented by a gaussian fit whose maximum wavelength and spectral bandwidth follows the values shown in Table 1. An example of a fitted DUV spectrum is shown in Fig. 9a.

**Table 1:** Gaussian Parameters taken into account to fit the fluorescence emission spectrum of the endogenous fluorophores excited in DUV range (275 nm) and in NIR range (810 nm and 890 nm).

	Maximum wavelength (nm)	Spectral bandwidth (nm)
**DUV fluorophores**		
Tyrosine	301–311	0–50
Tryptophan	335–345	0–10
Collagen	380–420	0–50
NADH	430	0–60
**NIR fluorophores**		
SHG (890 nm only)	440–450	0–5
NADH free	460–470	45–50
NADH bound (810 nm only)	443–445	40–48
FAD	520–530	30–50
Lipopigments	570–600	0–180
Porphyrins I	615–630	0–10
Porphyrins II	675–690	0–10

Through this spectral fitting process, the Integral proportion (Int) of each fluorophore emission can be determined. It’s defined as the ratio of the integral under the emission curve of the fluorophore contribution on the integral under the total fitted spectrum curve. Rationing the different calculated integral proportion, we were able to extract three different DUV molecular ratios:

1 $${\text{Tryp}}/{\text{Tyr}} = {\text{Tryptophan - Int}}/{\text{Tyrosine - Int}}$$

2 $${\text{Tryp}}/{\text{NADH}} = {\text{Tryptophan - Int}}/{\text{NADH - Int}}$$

3 $${\text{Tryp}}/{\text{coll}} = {\text{Tryptophan - Int}}/{\text{Collagen - Int}}$$

#### NIR spectral analysis

NIR Spectral images were extracted first using the image processing software “FIJI” and then processed using a homemade Matlab interface for molecular ratios extraction and for phasor plot analysis.

For spectral phasor analysis, the fluorescence spectrum of each pixel in each spectral image was recovered. Two coordinates can be extracted through the Fourier transform of each spectrum: the real and imaginary parts. These two coordinates were used to plot the corresponding “phasor” (or count) of the pixel in a histogram plot known as the phasor plot. The grid of the phasor plot covers the maximum emission peaks of the spectrum from the spectral range of 380–790 nm by 10 nm steps, and the spectral width varies from 20 to 100 nm by steps of 20 nm each. Therefore, one total histogram can be obtained for each sample, and afterwards all the histograms belonging to the samples of the same type were added together in order to plot global type histogram.

In addition, standard phasor histograms of NADH and FAD, measured through standard fluorophores solutions (1 mM in Tris Buffer, pH = 8.5 for NADH and 1 mM in PBS, pH = 7.4 for FAD), were added to each global type histogram and marked in red on the grid in order facilitate the tracking of the tissues emission peaks.

For NIR molecular ratios extraction, the same spectral fitting technique used for DUV spectral data processing was applied to fit the NIR spectral data. The mean fluorescence spectrum of all pixels in each spectral image was fitted to extract the integral proportion of each fluorophore emission. At 810 nm excitation wavelength, six fluorophores were chosen and fitted by Gaussian curves through a literature review^18^: NADH free, NADH protein bound, FAD, lipopigments, porphyrin I and porphyrins II. While at 890 nm excitation wavelength, the NADH bound was removes and replaced by SHG emission. The Gaussian parameters used for these fluorophore emission fitting is recapitulated in Table 1, NIR section. An example of two fitted NIR spectrum is shown in Fig. 9a,b. Rationing the different calculated integral proportion (Int) of these fluorophores, we were able to extract three different NIR molecular ratios:

4 $${\text{Redox}}\,{\text{ratio}} = {\text{FAD - Int}}/\left( {{\text{NADH - free - Int}} + {\text{NADH - bound - Int}}} \right) + {\text{FAD - Int}}$$

5 $${\text{PN}}\,{\text{ratio}} = \left( {{\text{PorphyrinsI - Int}} + {\text{PorphyrinsII - Int}}} \right)/\left( {{\text{NADH - free - Int}} + {\text{NADH - bound - Int}}} \right)$$

6 $${\text{LP}}\,{\text{ratio}} = {\text{Lipopigments - Int/}}\left( {{\text{PorphyrinsI - Int}} + {\text{PorphyrinsII - Int}}} \right)$$

### FLIM analysis

FLIM acquisition were performed using 810 nm as excitation wavelength. The acquired data were extracted through Symphotime software (PicoQuant, GmbH, Berlin, Germany), then a Matlab interface was used to treat these data.

For protein-bound NADH lifetime, the channel 1 FLIM images were selected where several region of interest (ROI) were chosen in each image. In each ROI, the decay curves of its pixels were averaged and adjusted by a bi-exponential fit. To get an acceptable fit, two criteria were taken into account: (1) having χ^2^-values of around 1.0 (χ^2^ range 0.8–1.2) and (2) the residuals had to be randomly distributed around 0 within the intervals + 4 and − 4.

For free and protein bound FAD, the channel 2 FLIM image were selected where the phasor approach was used to extract their lifetime values. For each sample, FLIM measurements were performed on a 3 × 3 mosaic images (9 images). Each image was extracted and treated using “FIJI” software, the size of each image was reduced from 512 × 512 pixels to 16 × 16 pixels. So each new reduced pixel presents a real size of 32 × 32 = 1024 pixels. The decay curves of each 1024 pixels were added together to obtain one decay curve I(t). Each decay curve I(t) is represented in a graphical view by a unique vector having its unique location, called phasor (or count) in the “phasor plot”^42–44^.

Then each decay curve I(t) is converted in a Cartesian plot into two coordinates using the Eqs. (7) and (8) below:

7 $${\mathrm{S}}_{\mathrm{i}}\left(\upomega \right)=\underset{0}{\overset{\infty }{\int }}\mathrm{I}\left(\mathrm{t}\right).\mathrm{cos}\left(\mathrm{\omega t}\right).\mathrm{dt }/\underset{0}{\overset{\infty }{\int }}\mathrm{I}\left(\mathrm{t}\right).\mathrm{dt}$$

8 $${\mathrm{G}}_{\mathrm{i}}\left(\upomega \right)=\underset{0}{\overset{\infty }{\int }}\mathrm{I}\left(\mathrm{t}\right).\mathrm{sin}\left(\mathrm{\omega t}\right).\mathrm{dt }/\underset{0}{\overset{\infty }{\int }}\mathrm{I}\left(\mathrm{t}\right).\mathrm{dt}$$

The x and y coordinates of each phasor are represented by Si(ω) and Gi(ω) while the index “i” refers to a pixel in the reduced image; The laser repetition angular frequency is represented by w and it’s related to the sampling period (Ts) and to the signal length (L) through the Eq. (9) below:

9 $$\upomega =2\uppi /\mathrm{L}.\mathrm{Ts}$$

For each reduced pixel, we kept Si(ω), Gi(ω) and Mi: the normalized integration under the decay curve. These three sets of numbers provided the phasor histogram of the initial reduced 3 × 3 mosaïc image where each reduced pixel of the FLIM image present a count in the phasor plot histogram.

After this procedure, phasor counts of all the samples belonging to the same type were grouped to plot the global phasor histogram for each tissue type where the local maxima of the histogram were localized in order to draw the best fitting line. The two intersections points between the circle segment and this fitting line are related to the two fluorescence FAD components lifetime values^43,44^.

Another procedure was performed in order to calculate, for each tissue type, the long lifetime intensity fraction (LLIF) of NADH and FAD. This parameter is calculated in order to determine the fluorescence fraction emitted by each molecule component (free and protein-bound) presented in a FLIM image, so to track the emission changes of these two components with the tissue grade.

For that, we selected together all channel 1 (NADH) FLIM images belonging to the three tissue types, where a global molecule phasor histogram was plotted used these selected images. The local maxima of this histogram was localized and the fitting line was drawn. Then, for each tissue type, a projection on the line of its phasor counts was performed to calculate the (LLIF) histogram of NADH^44^. The same procedure was also applied on all channel 2 (FAD) FLIM images to calculate the LLIF histogram of FAD.

### Collagen 3D discrimination algorithm

Extracted from DUV and NIR spectral fitted data, three parameters related to collagen emission: SHG integral proportion (SHG-Int) of 890 nm NIR spectra, collagen integral proportion (Coll-Int) of DUV spectra and normalized collagen maximum peak (Coll-Peak) were combined and presented as a 3D-scatter plot for the three groups of tissues (Fig. 6). Each point in the scatter cloud has SHG-Int as X axis coordinate, Coll-Peak as Y coordinate and Coll-int as Z axis coordinated. The scatter cloud of each tissue was approached by a Gaussian ellipsoid using the mean and the standard deviation as parameters for the covariance with the ellipse to cover 60% of the total probability mass. Two discrimination tests were conducted on 10 control samples, 7 grade I meningioma samples and 8 grade II meningioma samples, where the sensitivity (Se) and the specificity (Sp) of the discrimination criteria were calculated using the Eqs. (10) and (11) cited below:

10 $${\text{Se}} = {\text{TP}}/\left( {{\text{TP}} + {\text{FN}}} \right)$$

11 $${\text{Sp}} = {\text{TN}}/\left( {{\text{TN}} + {\text{FP}}} \right)$$

The first test was realized between control ellipsoid and tumor ellipsoid using 2.5 value of SHG-Int as threshold to discriminate control from tumor tissues, where:

TP: True Positive, defined as tumoral tissue classified as tumoral.

FP: False Positive, defined as control tissue classified as tumoral.

TN: True Negative defined as control tissue classified as control.

FN: False Negative, defined as tumoral tissue classified as control.

The second test was realized between grade I meningioma ellipsoid and grade II meningioma ellipsoid using 22 value of SHG-Int as threshold to discriminate grade I from grade II samples, where:

TP: True Positive, defined as grade II tissue classified as grade II.

FP: False Positive, defined as grade I tissue classified as grade II.

TN: True Negative defined as grade I tissue classified as grade I.

FN: False Negative, defined as grade II tissue classified as grade I.
